# Drug Survival of Upadacitinib and Predicting Factors of Discontinuation in Adult Patients Affected by Moderate-to-Severe Atopic Dermatitis: An Italian Multicenter Analysis

**DOI:** 10.3390/jcm13020553

**Published:** 2024-01-18

**Authors:** Elena Pezzolo, Michela Ortoncelli, Silvia Mariel Ferrucci, Mario Bruno Guanti, Donatella Schena, Maddalena Napolitano, Mariateresa Rossi, Caterina Foti, Domenico D’Amico, Giuseppe Fabrizio Amoruso, Pietro Morrone, Simone Ribero, Francesca Barei, Matteo Biagi, Enrico Pascucci, Cataldo Patruno, Piergiacomo Calzavara Pinton, Paolo Romita, Luigi Gargiulo, Alessandra Narcisi, Luigi Naldi

**Affiliations:** 1 Dermatology Unit, Ospedale San Bortolo, 36100 Vicenza, Italy; 2 Study Centre of the Italian Group for Epidemiologic Research in Dermatology (GISED), 24128 Bergamo, Italy; 3 Dermatology Clinic, Department of Medical Sciences, University of Turin, 10124 Turin, Italy; 4 Dermatology Unit, Fondazione IRCCS Ca’ Granda Ospedale Maggiore Policlinico, 20122 Milan, Italy; 5 Department of Dermatology, University of Modena and Reggio Emilia, 41100 Modena, Italy; 6 Section of Dermatology and Venereology, Department of Medicine, University of Verona, 37100 Verona, Italy; 7 Department of Clinical Medicine and Surgery, University of Naples Federico II, 80131 Naples, Italy; 8 Dermatology Department, University of Brescia, 25123 Brescia, Italy; 9 Unit of Dermatology, Department of Precision and Regenerative Medicine and Jonian Area, University of Bari Aldo Moro, 70121 Bari, Italy; 10 UOC Dermatologia, AOU “R. Dulbecco”, Ospedale ‘A. Pugliese’, 88100 Catanzaro, Italy; 11 UOC Dermatologia, Azienda Ospedaliera Cosenza, 87100 Cosenza, Italy; 12 Department of Health Sciences, Magna Graecia University, 88100 Catanzaro, Italy; cataldo.patruno@unicz.it; 13 Department of Biomedical Sciences, Humanitas University, 20090 Milan, Italy; 14 Dermatology Unit, IRCCS Humanitas Research Hospital, 20089 Milan, Italy; alessandra.narcisi@humanitas.it

**Keywords:** atopic dermatitis, drug survival, pathogenesis, type 2 inflammation, Th2 response, treatment, upadacitinib

## Abstract

**Background:** Limited real-world data are available on upadacitinib drug survival in patients with atopic dermatitis (AD). **Objectives:** To investigate upadacitinib drug survival, and the reasons and predictors of drug discontinuation in AD patients. **Methods:** All consecutive patients aged 18–75 years, affected by moderate-to-severe AD, and treated with upadacitinib for more than 1 month at dermatological clinics were included during November 2020–August 2023. Upadacitinib survival was investigated through Kaplan–Meier survival analysis and the predictors through multivariable logistic regression analysis. **Results:** Overall, 325 adult AD patients (mean (SD) age, 38.6(15.6) years) had a 1-year and 1.5-year upadacitinib drug survival of 91.5% and 80.2%, respectively. The main reasons for drug discontinuation (25/325, 7.7%) were adverse events (4.9%), including cutaneous or infectious diseases (1.5%), such as acne and herpes zoster; blood test changes (1.2%), including hypercholesterolemia, creatine phosphokinase or liver enzyme elevation, and lymphopenia; urinary or respiratory infections (0.9%); deep venous thrombosis (0.3%); malignancies (0.3%); loss of consciousness (0.3%); and arthralgias (0.3%); followed by ineffectiveness (0.6%). No specific characteristic was significantly associated with an increased risk of upadacitinib discontinuation. **Conclusions:** Our findings show that upadacitinib was effective in moderate-to-severe AD after more than 1 year of continuous treatment but point to the need for clinical and laboratory monitoring of patients.

## 1. Introduction

Atopic dermatitis (AD) is a chronic inflammatory dermatosis that is associated with a significant burden on patients and family members [1,2]. AD continues to exhibit a high multi-dimensional burden of disease. Therefore, efficacious treatments to control its activity and to improve patient well-being, reducing the economic cumulative burden of the disease, are needed [3]. Several topical and conventional systemic immunosuppressant treatments have traditionally been used in AD, such as on-label steroids and cyclosporin A, or off-label methotrexate, azathioprine, and mycophenolate mofetil [4]. Recent advances in AD pathogenesis have introduced more targeted treatments, such as the biological agents dupilumab, lebrikizumab, and tralokinumab, and the inhibitors of Janus Kinase (JAK) abrocitinib, baricitinib, and upadacitinib [5,6]. Phase 3 randomized clinical trials (RCTs) and real-world studies have proven the long-term effectiveness and safety of dupilumab and tralokinumab for the management of moderate-to-severe AD [7,8,9,10,11,12,13,14]. However, AD inefficacious response or onset of specific adverse events (AEs), such as conjunctivitis or facial and neck redness, have been reported in 31%, 4–17%, and 11% of dupilumab-treated patients, respectively [15,16,17,18,19,20]. Likewise, injection site reactions and conjunctivitis have been described in 2% to 13.1% of tralokinumab-treated patients [21,22].

JAK inhibitors show different effectiveness and safety, owing to their varying grades of signal suppression along the JAK-STAT pathway. Among drugs targeting JAK-1, the European Medicines Agency (EMA) has recently approved upadacitinib to treat adult and adolescent patients affected by moderate-to-severe AD [23]. In RCTs, the effectiveness and safety of upadacitinib in treating moderate-to-severe AD have been well documented through daily doses of 15 or 30 mg, either when given alone or in combination with topical corticosteroids [24,25]. In addition, in a head-to-head trial with dupilumab, upadacitinib was shown to be superior to dupilumab in the treatment of patients with AD, with a significantly higher number of patients in the upadacitinib treatment group reaching all primary and secondary outcomes [26]. The efficacy and safety of upadacitinib has also been confirmed in real-life settings [27,28,29,30]. To the best of our knowledge, no real-world studies have analyzed the drug survival of upadacitinib together with predictive factors of discontinuation in AD patients. Drug survival represents a comprehensive outcome, summarizing different aspects of treatment including patient satisfaction, efficacy, safety, and tolerability. We investigated upadacitinib drug survival up to 1.5 years of follow-up and examined reasons for drug discontinuation and factors predicting discontinuation in adult patients affected by moderate-to-severe AD in a multicenter Italian real-world setting.

## 2. Materials and Methods

### 2.1. Study Design

This study was planned with a retrospective design. All consecutive 18–75-year-old patients affected by moderate-to-severe AD receiving therapy with upadacitinib at eleven hospital-based dermatology departments (eight university-based) in Italy and followed up for more than 1 month were included in our study. The first case receiving upadacitinib was registered in November 2020; the data lock took place in August 2023. Since the study protocol was conducted in accordance with standard clinical practice, institutional review board approval was not required. Each patient gave written informed consent to have their data collected during routine clinical practice, i.e., demographics and clinical parameters, included in this retrospective study. Our study was conducted in compliance with the ethical principles of the Helsinki Declaration. Data collection and management observed applicable rules, regulations, and directives concerning patient protection, such as patient privacy. Seventy-eight (24%) patients included in this study received upadacitinib through the national compassionate use program managed by the Italian Medical Agency AIFA [30]. In this compassionate use program, patients aged 18–75 years affected by moderate-to-severe AD who were resistant or manifested intolerance or a contraindication to conventional treatments for AD received either upadacitinib 15 or 30 mg according to the clinician’s choice and after a proper washout from previous therapies [30].

All patients enrolled in this study received a baseline dose of upadacitinib 15 or 30 mg orally and applied daily emollients, while topical medium- to very-high-potency corticosteroids or calcineurin inhibitors were utilized as required. Patients’ demographic and clinical information at baseline included residential area, age, gender, height, weight, AD features (i.e., age at AD onset, AD phenotypes, distribution of cutaneous lesions, AD severity, presence of atopic comorbidities, immunosuppressive drug history, and use of topical immunosuppressive therapy at baseline), and upadacitinib therapy (time duration, discontinuation, reasons for discontinuation, and AEs). Age at the beginning of upadacitinib treatment was classified into (I) less than 65 years and (II) 65 years or older. AD phenotypes were divided into (I) classical type, (II) portrait AD, (III) hand eczema, (IV) erythroderma, (V) nummular type AD, and (VI) prurigo nodularis-like AD (PN-like AD). Patients were considered as using immunosuppressive agents at baseline when oral corticosteroids, cyclosporine, methotrexate, or other immunosuppressants for AD had been taken within 1 month before starting upadacitinib treatment, or, in the case of targeted biologic treatments, when they had been taken within 3 months or 5 half-lives, whichever was longer, before the start of upadacitinib treatment.

The severity of AD was measured through the Eczema Area and Severity Index (EASI) on a range of 0–72, through the Numeric Rating Scale (NRS) itch and sleep loss on a range of 0–10, and through the Dermatology Life Quality Index (DLQI) on a range of 0–30. Moreover, information on the duration of the therapy and the reason for upadacitinib withdrawal was collected. Safety evaluation included a physical exam and laboratory analyses, such as full blood count, liver and renal function tests, glucose, creatine phosphokinase, common coagulation pathway (i.e., prothrombin time, activated partial thromboplastin time, and international normalized ratio), QuantiFERON blood test, and hepatitis B and hepatitis C screening. AEs were classified as any expected and unexpected alterations to physiological condition or blood test changes reported by the physicians up to the end of treatment. Clinical outcomes were reported at baseline, week 4, week 16, week 32, week 52, and week 72.

### 2.2. Statistical Analysis

Descriptive information was set up through mean and standard deviation (mean ± SD), or median and interquartile (IQR), or absolute numbers and percentages. Upadacitinib survival was investigated through Kaplan–Meier survival analysis to calculate the risk of, and time to, drug withdrawal [31]. A separate analysis of three upadacitinib survival curves was performed: overall upadacitinib withdrawal, withdrawal owing to inefficacy, and withdrawal owing to AEs. Causes for overall upadacitinib withdrawal were classified as ineffectiveness, AEs, patient’s choice, pregnancy wish, or SARS-CoV-2 infection. Patients were censored if they were receiving upadacitinib up to the time of study close in August 2023, if the cause of discontinuing upadacitinib was not related to effectiveness or safety (e.g., pregnancy wish), or in case of loss to follow-up. For all participants, exclusively the first course of upadacitinib was evaluated; a single course was considered if the treatment was temporarily withdrawn for a period of less than 90 days. Study outcomes across groups were compared between baseline and weeks 4, 16, 32, 52, and 72 through the Wilcoxon rank test.

Parameters such as age, gender, BMI, age at onset of AD, AD course, AD clinical phenotype, allergic asthma, allergic rhinitis, allergic conjunctivitis, non-atopic comorbidities, immunosuppressive drug history, delta EASI, and delta itch after 4 weeks of treatment were selected as possible predictive factors of upadacitinib drug survival. The delta EASI was divided into (I) patients who did not respond to upadacitinib at 1 month when delta EASI ≥ 0 (corresponding to an absence of response or aggravation of AD at 1 month of therapy compared with baseline) and (II) patients who responded when delta EASI <0. Early-onset AD corresponded to AD developing before 18 years of age. All potential predictive factors of upadacitinib withdrawal were included in an adjusted multivariable regression analysis to assess the interactions among all of the parameters. Since the cases of upadacitinib withdrawal due to inefficacy and/or AEs were only a few for the number of predictive factors to be assessed, we could not perform a sub-analysis of these predictive factors separately for the reason for drug discontinuation. Two-sided *p* values <0.05 were considered statistically significant. Analysis of the data was performed using IBM SPSS Statistics (version 26, IBM Corporation, Armonk, NY, USA).

## 3. Results

### 3.1. Study Population

In total, 325 adult patients with moderate-to-severe AD (mean (SD) age, 38.6 (15.6) years) were enrolled at the beginning of therapy with upadacitinib. Overall, 198 patients (60.9%) were male and 45 patients (13.8%) applied topical immunosuppressants at the beginning of therapy. A total of 266 patients (81.8%) were seen in centers in Northern Italy, while 59 (18.2%) attended centers in Southern Italian regions. The first occurrence of AD was more frequent in childhood (0–11) (58.2%, 189/325) and adulthood (18–64) (28.9%, 94/325), while less frequent during adolescence (11–18) and in the elderly (>64). The flexural phenotypes of AD, mainly associated with head and neck and/or hand AD, were observed in 72.6% (236/325) of the patients; other clinical AD phenotypes included nummular eczema (10.5%, 34/325), PN-like AD (4.6%, 15/325), and erythrodermic patterns (4.6%, 15/325). Most of the patients had received previous treatment with more than two systemic medications, with dupilumab prescribed in 185 out of 325 (56.9%) patients; they had a 2- to 4-week wash-out period from previous monoclonal antibody therapy. The baseline average EASI score was 22.6 (11.3), and patients reported average NRS itch and sleep loss scores of 7.6 (2.4) and 6.8 (2.6), respectively, and a DLQI of 14.6 (8.2).

At the time of data lock, in August 2023, 90.8% of patients (*n* = 295/325) were still using upadacitinib. Twenty-five patients (7.7%) had withdrawn upadacitinib, while another five patients were lost to follow-up. Two patients (0.6%) had withdrawn upadacitinib due to inefficacy. Sixteen patients (4.9%) discontinued upadacitinib due to AEs. Two patients (0.6%) withdrew upadacitinib due to pregnancy, and five patients due to different causes (1.5%), such as the patient’s personal decision and SARS-CoV-2 infection. The observation periods and the median interquartile ranges (IQRs) of continuous upadacitinib therapy up to withdrawal are reported in Table 1.

The characteristics of the study population differentiated by the reason for upadacitinib discontinuation are presented in Table 2. Twelve patients (3.7%) had an absence of improvement or an aggravation of AD at month 1 (average EASI score increment of 13.4%) in comparison with baseline; they were classified as nonresponders at month 1. Patients who responded at month 1 (313 out of 325, 96.3%) showed an average EASI decrease of 71.4%; nearly half of them (49.8%) achieved an EASI of ≤7, and 35.4% achieved an EASI of ≤3. At month 1, the average percentage reduction in NRS itch score was 64.5% (from 7.6 at baseline to 2.7 at month 1); 63.4% (206 out of 325) of patients reached a ≥ 4-point increase in the NRS itch score compared to baseline, and 35.4% (115 out 325) achieved an NRS itch score of ≤1. We performed the analysis using the time point of 1 month because, through Spearman statistics, the EASI scores between week 4 and week 16 were strongly correlated (0.81) in nonresponder patients.

**Figure 1:**
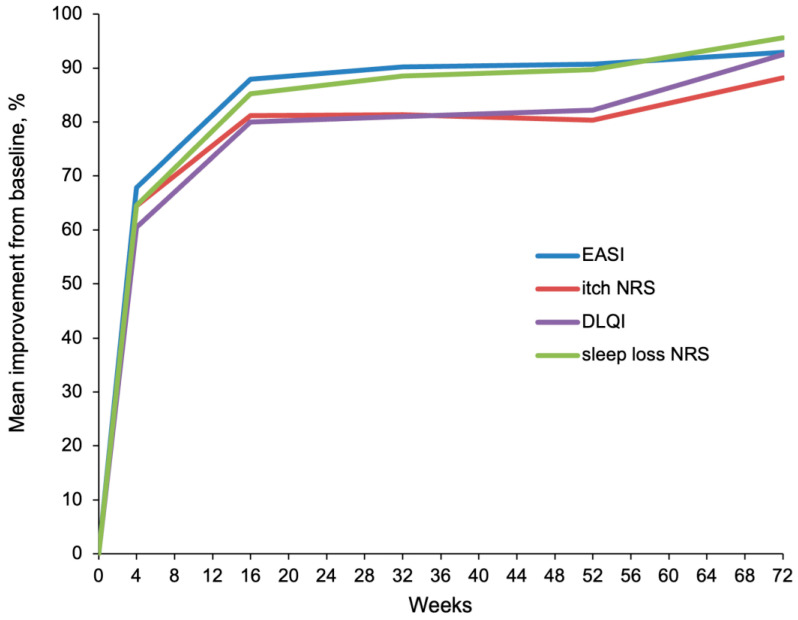
Mean percentage improvement in EASI, NRS itch and sleep loss, and DLQI from baseline through 52 and 72 weeks of treatment with upadacitinib.

The improvement observed in the first month was maintained through 72 weeks of continuous treatment with upadacitinib with an additional increase in response. After 16 weeks of treatment, response rates for EASI-75 and EASI-90 were 87.5% (281 out of 321) and 62.3% (200 out of 321), respectively, while response rates for ≥4-point improvement on the NRS itch and for NRS itch 0/1 were comparable to those detected at week 4. Since week 4 EASI, NRS itch and sleep loss, and DLQI scores continually improved up to week 52 (*n* = 179 patients) and week 72 (*n* = 78 patients) (Figure 1). A constant reduction in the average EASI score from baseline (22.6) up to weeks 52 (2.1) and 72 (1.6) was observed; the average rate of reduction was 90.7% and 92.9%, respectively. The average NRS itch score decreased from baseline (7.6) by 80.3% up to week 52 (1.5), and by 88.2% up to week 72 (0.9). The average NRS sleep loss score reduced from baseline (6.8) by 89.7% up to week 52 (0.7), and by 95.6% up to week 72 (0.3). The average decrease of DLQI from baseline (14.6) up to week 52 (2.6) and week 72 (1.1) were 82.2% and 92.5%, respectively. All these decreases were statistically significant (*p* value <0.01).

After 52 weeks of continuous treatment with upadacitinib, EASI-50 was achieved by 94.4% (*n* = 169/179) of patients, EASI-75 by 88.8% (*n* = 159/179) of patients, EASI-90 by 73.7% (*n* = 132/179) of patients, and EASI-100 by 40.2% (*n* = 72/179) of patients. At week 72, EASI-50, EASI-75, EASI-90, and EASI-100 were reached by 97.4% (*n* = 76/78), 91.0% (*n* = 71/78), 84.6% (*n* = 66/78), and 62.8% (*n* = 49/78) of patients, respectively.

The AEs during treatment with upadacitinib, overall and leading to drug withdrawal, are reported in Table 3. The main reason for the discontinuation of upadacitinib due to AEs was cutaneous or infective AEs (*n* = 5 (1.5%)), including herpes zoster in three patients (0.9%) who had not received the herpes zoster vaccination, papulopustular acne (0.3%), and psoriasiform lesions (0.3%). All sixteen patients who discontinued treatment owing to AEs had a history of previous use of immunosuppressants and an EASI score of severe AD, severe or very severe NRS itch and sleep loss scores, and a DLQI score corresponding to a severe or very severe impact on quality of life. Other reasons for discontinuation of upadacitinib were blood test changes (*n* = 4, 1.2%), including severe hypercholesterolemia (0.3%), increased values of creatine phosphokinase (CPK) (0.3%), lymphopenia (0.3%), and increased liver enzymes (0.3%), followed by urinary (0.3%) or respiratory tract (0.6%) infections (*n* = 3, (0.9%)), deep venous thrombosis (0.3%), malignancies (0.3%), loss of consciousness (0.3%), and arthralgias (0.3%). The patient with anal cancer and the patient with deep venous thrombosis permanently discontinued treatment after 4 and 8 weeks, respectively. Anal cancer developed within 1 month of therapy with upadacitinib in a male patient aged 70 years with diabetes mellitus, whereas deep venous thrombosis occurred within 2 months of treatment with upadacitinib in a male hypertensive patient aged 73 years. A total of 20 (6.1%) patients temporarily discontinued upadacitinib, and 16 (80%) of them maintained responsiveness.

### 3.2. Drug Survival Analysis and Regression Analysis

**Figure 2:**
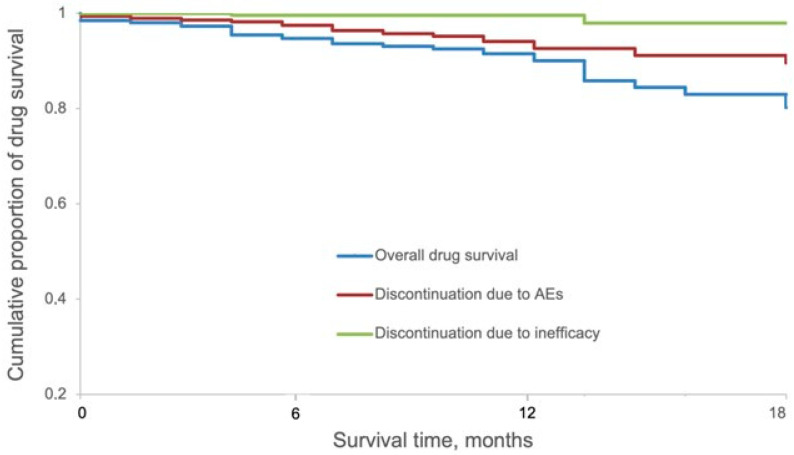
Kaplan–Meier survival curves for overall 1.5-year drug survival of upadacitinib split according to reasons of discontinuation (ineffectiveness or AEs).

After 1 and 1.5 years of continuous treatment with upadacitinib, the overall (i.e., owing to all reasons of discontinuation) drug survival was 91.5% and 80.2%, respectively. The drug survival of upadacitinib owing to AEs was 94.1% and 89.6% after 1 and 1.5 years, respectively. The drug survival with ineffectiveness as an event was 99.6% and 97.9% after 1 year and 1.5 years, respectively (Figure 2).

The univariable analysis showed that no specific patient characteristic, such as age, gender, BMI, baseline EASI or itch, delta EASI or delta itch after 4 weeks of treatment, prior immunosuppressant therapy, age of AD onset, or clinical phenotype of AD was significantly associated with an increased risk of upadacitinib discontinuation. The same features were not associated with an increased risk for upadacitinib discontinuation when adjusted in a multivariable analysis.

## 4. Discussion

By relying on survival analysis, we found an overall upadacitinib drug survival rate of 91.5% and 80.2%, after 1 year and 1.5 years of treatment, respectively. Separating the survival analysis by the cause of drug withdrawal, after 1 and 1.5 years the drug survival of upadacitinib owing to AEs was 94.1% and 89.6%, respectively, and the drug survival owing to inefficacy was 99.6% and 97.9%, respectively. We found that no single characteristic of the study population showed a significant association with drug discontinuation. Demographic and disease characteristics of our study population at baseline were comparable to those reported on upadacitinib in RCT [25].

The main reasons for discontinuation of upadacitinib, which occurred in 7.7% of patients, included AEs, ineffectiveness, and other reasons such as the patient’s personal decision, pregnancy wish, and SARS-CoV-2 infection. Among the AEs, cutaneous or infectious diseases were the principal cause of withdrawal, which occurred in 1.5% of the patients and included herpes zoster infection, papulopustular acne (also called Jakne), and psoriasiform lesions. They were followed by blood test changes (1.2%), such as severe hypercholesterolemia, lymphopenia, and CPK or increased liver enzymes; infections (0.9%), including respiratory and urinary tract infections; deep venous thrombosis; malignancies; and arthralgias. These AEs mainly occurred in the first months after drug initiation and developed in adulthood. The patient who discontinued upadacitinib due to a deep venous thrombosis was the male non-smoking patient aged 73 years with hypertension, who was recently reported by Chiricozzi et al. (Table 3) [32]. This patient was treated with a starting dose of 30 mg upadacitinib daily before the publication of the EMA recommendations to revise the specific dosage in old age to 15 mg once daily [32]. One case of anal cancer occurred in a 70-year-old male patient with diabetes mellitus within the first 4 weeks of treatment with upadacitinib 15 mg; however, it might be considered unrelated to upadacitinib, since the time of occurrence was very close to the time of treatment initiation (Table 3). Overall, three out of twenty (15%) patients aged ≥ 65 years who received upadacitinib withdrew from the treatment due to AEs, whilst none discontinued it due to inefficacy (Table 2). In our study, the re-treatment after discontinuation in those patients who temporarily discontinued upadacitinib (20 out of 325, 6.1%) was not associated with a loss of efficacy, maintaining responsiveness in 16 out of 20 (80%) cases [33].

Only two out of three hundred and twenty-five (0.6%) patients discontinued upadacitinib owing to ineffectiveness during the whole follow-up period of 72 weeks, hence proving its excellent long-term efficacy [34].

Rapid improvement of disease severity in terms of EASI and a ≥ 4-point improvement on the NRS itch score was obtained at the first follow-up visit at week 4 in 96% and 63.4% of patients, respectively. In line with the treat-to-target recommendations for AD by an international expert consensus [35], in our study, both early and late therapeutic goals, i.e., after 3 and 6 months, respectively, were achieved in a great proportion of patients (almost 88%) [35]. At week 16, a higher percentage of patients receiving upadacitinib 15 mg daily reached EASI-75 in our study, compared to those found in Measure Up 1 and Measure Up 2 clinical trials (87.5% vs. 70% and 60%, respectively) [25,26]. A contribution to the higher clinical improvements observed in our study may have been provided by the association with topical low-to-medium potency corticosteroids. However, in the AD Up trial, this association did not strengthen the effectiveness of upadacitinib (77.1% of EASI-75 response at week 16) [25,26].

Differences in clinical outcomes, such as EASI-90, between RCTs and our study were also observed. Indeed, 62.3% of our study population achieved EASI-90 after 16 weeks of treatment, compared to 53.1% and 42.4% of patients in the Measure Up 1 and 2 trials, respectively. Moreover, through 1 year of continuous treatment with upadacitinib, a slightly superior response rate of EASI-75 was found, compared to the one reported in Simpson et al.’s work (88.8% vs. 82.0% and 79.1%, respectively) [36]. Clinical improvements were associated with relevant responses across different dimensions, such as itch, sleep, and quality of life [37].

Notably, a higher proportion of patients reached DLQI 0/1 at week 16, as compared with week 4 (54.5% vs. 20%). This is in line with the consistent improvement in DLQI after 16 weeks of treatment in 41.1% of patients in the Measure Up 1 trial [36].

The comprehensive safety profile of upadacitinib was favorable in accordance with RCTs, exhibiting the following most frequent AEs: hypercholesterolemia, increase in liver enzymes, acne, herpes simplex/zoster infection, anemia, plasma CPK elevation, and upper respiratory tract infection. The greater proportion of these AEs were mild, temporary, and did not lead to upadacitinib discontinuation [38]. Most herpes zoster infections were nonserious and presented as circumscribed dermatoses; only in three cases did they lead to treatment withdrawal [38].

No studies to date have evaluated the long-term drug survival of upadacitinib and the associated predictive factors of discontinuation. To the best of our knowledge, only two recent Italian cohort studies, a 52-week retrospective study and an interim analysis through 48 weeks of observation [32,39], and a Belgian 54-week retrospective study [40], have evaluated the efficacy and safety profiles of upadacitinib in the long-term management of AD in a real-world setting. Our study was performed between November 2020 and August 2023, and provided novel information regarding the overall upadacitinib drug survival rate of 94.1% and 89% after 1 and 1.5 years of treatment. Moreover, in our analysis, we found that no clinical or demographic characteristic of the study population was significantly associated with an increased risk of upadacitinib discontinuation.

The retrospective design of our study represents its main limitation. Moreover, we could not perform a sub-analysis of the predictors of upadacitinib survival by the specific reasons for discontinuation, such as ineffectiveness and AEs, because of the limited number of patients included and drug interruptions.

## 5. Conclusions

Our findings show that upadacitinib had a good survival rate after more than 1 year of continuous treatment in patients with AD. Upadacitinib provided rapid and long-term effectiveness, but also pointed to the need for a follow-up of patients. However, additional information on a greater cohort of patients followed in the long-term will help to support these preliminary findings and to investigate the role of potential predictive factors for treatment discontinuation.

## Figures and Tables

**Table 1:** Number of patients and duration of treatment according to follow-up status and reasons for discontinuation at data lock.

Status of Upadacitinib Treatment by Data Lock	No. (%)	Duration of Upadacitinib Treatment (Median (IQR)), Weeks
Active	295 (90.8)	12 (8–13.5)
Discontinued	25 (7.7)	12 (8–12)
Lost to follow-up	5 (1.5)	4 (4–8)
**Reasons for discontinuation**		
Ineffectiveness	2 (0.6)	8 (6–10)
Adverse effects	16 (4.9)	10 (7–12)
Patient’s choice	3 (0.9)	12 (12–12)
Pregnancy wish	2 (0.6)	15 (13.5–16.5)
SARS-CoV-2 infection	2 (0.6)	15 (13.5–16.5)

**Table 2:** Study population characteristics differentiated by the main reasons for upadacitinib withdrawal.

Characteristics	No.	Ineffectiveness	AEs
Total (%)	325 (100)	2 (100)	16 (100)
Sex (%)			
Female	127 (39.1)	1 (50.0)	7 (43.8)
Male	198 (60.9)	1 (50.0)	9 (56.2)
Age, years, mean (SD)	38.6 (15.6)	21.5 (0.7)	43.4 (17.3)
<65 years	305 (93.8)	2 (100)	13 (81.2)
≥65 years	20 (6.2)	0 (0.0)	3 (18.8)
BMI, mean (SD)	24.2 (3.6)	23.7 (3.1)	23.9 (5.4)
Age at AD onset (%)			
Early-onset (<18 years)	231 (71.1)	2 (100)	8 (50.0)
Late-onset	94 (28.9)	0 (0.0)	8 (50.0)
AD phenotype (%)			
Classical	236 (72.6)	2 (100)	11 (68.8)
Portrait	17 (5.2)	0 (0.0)	1 (6.2)
Hand eczema	8 (2.5)	0 (0.0)	1 (6.2)
Erythroderma	15 (4.6)	0 (0.0)	0 (0.0)
Nummular	34 (10.5)	0 (0.0)	2 (12.6)
PN-like	15 (4.6)	0 (0.0)	1 (6.2)
Immunosuppressive drug history (%)			
1 prior immunosuppressive drug	83 (25.5)	0 (0.0)	8 (50.0)
≥2 prior immunosuppressive drugs	242 (74.5)	2 (100)	8 (50.0)
Prior biologics	185 (56.9)	1 (50.0)	10 (62.5)
Atopic comorbidities (%)	47 (14.5)	0 (0.0)	7 (43.8)

Abbreviations: AD, atopic dermatitis; AEs, adverse effects; BMI, body mass index; EASI, Eczema Area and Severity Index; NRS, Numerical Rating Scale; PN, prurigo nodularis.

**Table 3:** AEs reported during treatment overall and those leading to drug withdrawal, with duration of upadacitinib treatment up to withdrawal.

AEs	Overall No. (%)	Patients’ Age Mean (Range), Years	Leading to Withdrawal No. (%)	Duration of Upadacitinib Treatment Up to Withdrawal Median (IQR), Weeks
Cutaneous or infective	42 (12.9)	35.9 (18–73)	5 (1.5)	12 (4–18)
Herpes zoster	10 (3.1)	37.4 (18–55)	3 (0.9)	18 (15–18)
Herpes simplex	6 (1.8)	33.0 (18–56)	0 (0.0)	0 (0)
Papulopustular acne	24 (7.4)	35.9 (19–73)	1 (0.3)	4 (4–4)
Psoriasiform lesions	1 (0.3)	44.0 (44–44)	1 (0.3)	4 (4–4)
Seborrheic dermatitis	2 (0.6)	36.5 (21–52)	0 (0.0)	0 (0)
Blood test changes	55 (16.9)	41.2 (19–72)	4 (1.2)	10 (6.25–13.50)
Hypercholesterolemia	33 (10.2)	42.0 (19–66)	1 (0.3)	8 (8–8)
Increased CPK	5 (1.5)	31.7 (23–48)	1 (0.3)	1 (1–1)
Decreased WBC	2 (0.6)	38.0 (21–55)	1 (0.3)	12 (12–12)
Decreased RBC	1 (0.3)	68.0 (68–68)	0 (0.0)	0 (0)
Increased liver enzymes	9 (2.8)	38.7 (26–72)	1 (0.3)	18 (18–18)
Infections	7 (2.1)	34.6 (22–57)	3 (0.9)	12 (10–12)
Respiratory tract	4 (1.2)	36.0 (22–57)	2 (0.6)	12 (12–12)
Urinary tract	3 (0.9)	32.7 (26–40)	1 (0.3)	8 (8–8)
Deep venous thrombosis	1 (0.3)	73.0 (73–73)	1 (0.3)	4 (4–4)
Malignancies	1 (0.3)	70.0 (70–70)	1 (0.3)	4 (4–4)
Loss of consciousness	1 (0.3)	28.0 (28–28)	1 (0.3)	4 (4–4)
Arthralgias	1 (0.3)	30.0 (30–30)	1 (0.3)	8 (8–8)
Asthenia	4 (1.2)	42.0 (30–54)	0 (0.0)	0 (0)

Abbreviations: AEs, adverse events; CPK, creatine phosphokinase; WBC, white blood cells; RBC, red blood cells.

## Data Availability

The data that support the findings of this study are available from the corresponding author upon reasonable request.
